# Advancing the Pharmaceutical Potential of Bioinorganic Hybrid Lipid‐Based Assemblies

**DOI:** 10.1002/advs.201800564

**Published:** 2018-07-20

**Authors:** Junqing Wang, Angela Zhe Wang, Peng Lv, Wei Tao, Gang Liu

**Affiliations:** ^1^ State Key Laboratory of Molecular Vaccinology and Molecular Diagnostics & Center for Molecular Imaging and Translational Medicine School of Public Health Xiamen University Xiamen 361102 China; ^2^ Center for Nanomedicine and Department of Anesthesiology Brigham and Women's Hospital Harvard Medical School Boston MA 02115 USA; ^3^ Blood Cancer Cytogenetics and Genomics Laboratory Department of Anatomical and Cellular Pathology Prince of Wales Hospital The Chinese University of Hong Kong Shatin Hong Kong S.A.R. China; ^4^ State Key Laboratory of Cellular Stress Biology Innovation Center for Cell Biology School of Life Sciences Xiamen University Xiamen 361102 China; ^5^ The MOE Key Laboratory of Spectrochemical Analysis & Instrumentation College of Chemistry and Chemical Engineering Xiamen University Xiamen 361005 China

**Keywords:** cerasomes, lipid assemblies, metallosomes, metallosurfactants, silylated lipids

## Abstract

Bioinspired lipid assemblies that mimic the elaborate architecture of natural membranes have fascinated researchers for a long time. These lipid assemblies have gone from being just an imperative platform for biophysical research to a pharmaceutical delivery system for biomedical applications. Despite success, these organized nanosystems are often subject to the mechanical instability and limited theranostic capability without adding any inconvenient modifications. To reach their advanced pharmaceutical potential, various bioinorganic hybrid lipid‐based assembles, which provide new opportunities to synergistically complement and improve therapeutic/diagnostic potential of existing lipid‐based nanomedicine with distinct mechanisms containing inorganic embedded surfactants, have recently been developed.

## 1 Introduction

Major breakthroughs in the lipid nanotechnology field during the past 30 years have resulted in fruitful liposomal and other lipid‐based products available for clinical use.^1^ Their resulting interest in this field is due to these products' inherent biocompatibility, biodegradability, and their various structural characteristics (based on the preparation optimization and design of hydrophilic/hydrophobic moieties within the amphiphile units),^2^ which allow them to self‐organize to form liposomes,^3^ vesicles,^4^ micelles,^5^ bicelles,^6^ and liquid crystalline dispersions^7^ for diverse therapeutics delivery.^8^ However, these bilayer and acyl‐chain lipid products have not fulfilled their practical potential due to their insufficient morphological stability and inclusion leakage in vitro and in vivo.^9^ To address these drawbacks, various optimizations have been approached,^10^ which include surface coating with certain amphiphilic molecules (PEGylation; where PEG is polyethylene glycol) and cholesterols for prolonging circulation,^11^ enhancing elasticity using transferosome formulations for transdermal delivery,^12^ and improving bioavailability that employ drug–lipid conjugates.^13^ Although these strategies have significantly promoted the therapeutic potential of traditional lipid nanoparticles with increasingly broad applications, several concerns such as insufficient coverage of surface coating on phospholipid bilayers,^14^ and the morphological stability of lipid assemblies are likely to decrease after modulating composition, charge, and bilayer architecture.

**Figure 1:**
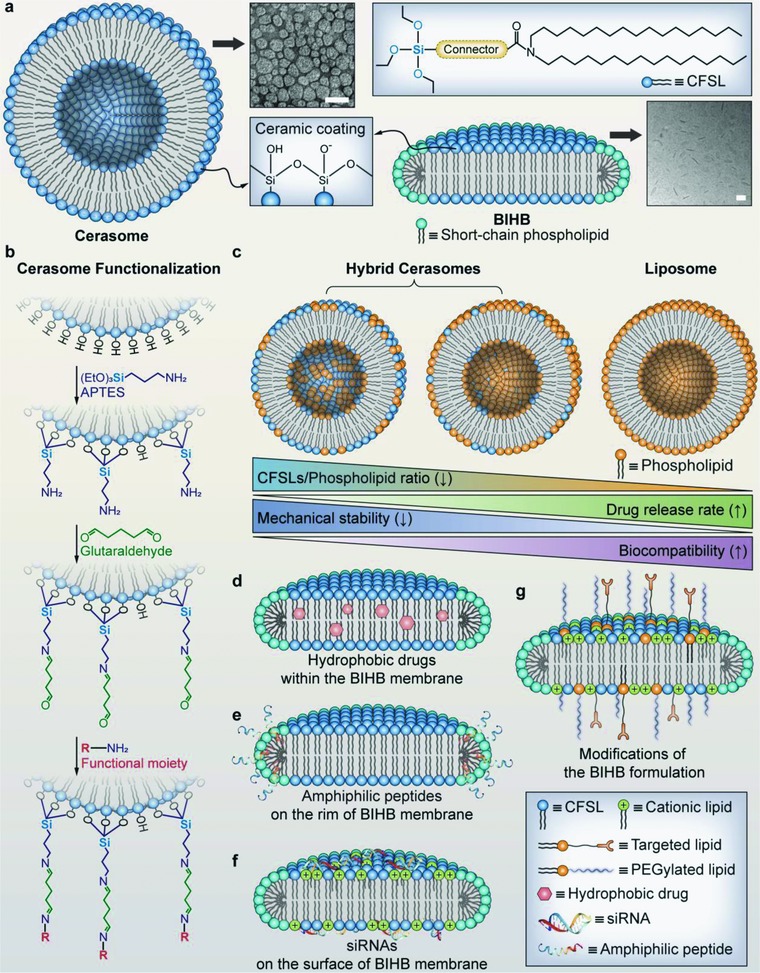
Toward bioinorganic hybrid lipid‐based assemblies. a) Schematic representation of general molecular structure and representative TEM images of cerasome and bioinorganic hybrid bicelles (BIHB). b) A schematic illustration of reported cerasome surface functionalization process, which initiated by silanization of 3‐aminopropyltriethoxysilane (APTES) molecules on cerasome surface, followed by Schiff‐base reaction between amine and aldehyde groups of glutaraldehyde.^28^ Then, functional moiety containing amine group was attached following the surface activation with glutaraldehyde. c) Effect of CFSLs/phospholipid ratio on drug release rate, mechanical stability, and biocompatibility of hybrid cerasomes. d–g) Structural and design strategies for therapeutic delivery using bioinorganic hybrid bicelles (BIHBs). Hydrophobic molecules can be embedded in bilayer membrane (d). Amphiphilic peptides can be attached on the curved rim‐region of the BIHB (e). siRNAs can be loaded on the surface of cationic lipid‐incorporated BIHBs (f). BIHBs can be surface functionalized to endow stealth effect by incorporate PEGylated lipids, and to promote active endocytosis by introducing targeting lipids, as well as to modulate the surface charge by doping cationic lipids (g). Abbreviation: CFSL: cerasome‐forming silylated lipid; (Scale bar = 50 nm). TEM images were reproduced with permission.^29^, ^30^ Copyright 2006, Nature Publishing Group; Copyright 2011, Royal Society of Chemistry.

As counterparts of organic nanosystems, inorganic or complexed nanomaterials commonly involves mesoporous silicon, iron oxides,^15^ noble nanometals, quantum dots,^16^ graphene‐like 2D nanomaterials^17^ and metal–organic frameworks,^18^ which provide unique physical properties, better morphological stability, and multifunctionality.^19^ However, many of them still suffer from poor biocompatibility and biodegradability. To this end, a family of artificial lipid‐based assemblies that integrate with inorganic building units, namely, bioinorganic hybrid lipid‐based assembles (BIHLAs) (**Figures** **1**a and 3a) such as cerasomes (ceramic hybrid liposome),^20^, ^21^ bioinorganic hybrid bicelles (BIHBs),^22^, ^23^ metallosomes,^24^ and clay–lipid biohybrid materials,^25^ which have progressively been recognized as novel theranostic nanostructures that integrate the advantages of both organic and inorganic nanomaterials but overcome their shortcomings. These novel nanostructures with desired functions can be considered as harmonized results of nanoarchitectonics strategy,^26^ a universal methodology that includes regulated atomic/molecular control, chemical modification, controlled physicochemical interactions, self‐assembly and self‐organization, as well as structural regulation of physical stimuli.^27^

This study describes the current state‐of‐the art information about the present and newly emerged BIHLAs (i.e., typically cerasomes, BIHBs, and metallosomes) for drug delivery and nanotheranostic applications. A critical highlight of the progression of their design, preparation, and functional modification is also described.

## 2 Cerasomes

**Figure 2:**
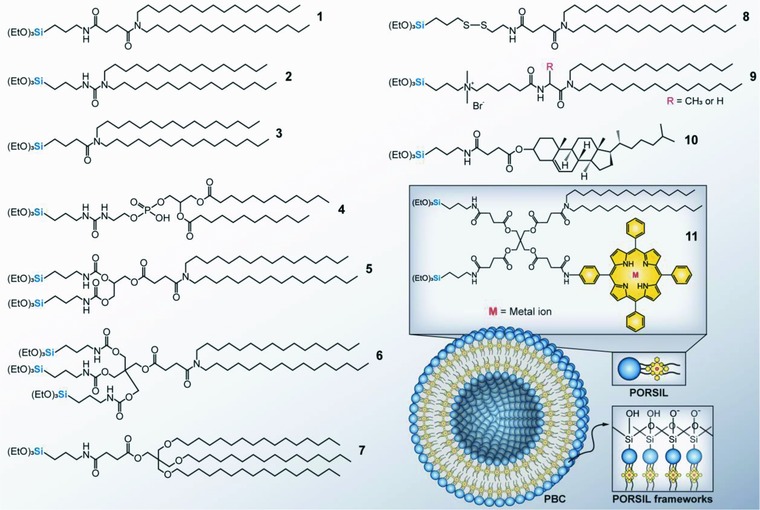
Survey of typical cerasome‐forming silylated lipids (CFSLs). CFSLs **1** is weakly negatively charged under physiological condition and was commonly used for in vivo drug delivery. CFSLs **2 and 3** containing a urea group and one peptide bond, respectively.^42^ CFSLs **4** is a biodegradable phospholipid with triethoxysilyl headgroup, which provide improved biocompatibility. CFSLs **5 and 6** bearing two to three triethoxysilyl heads were synthesized to tune the siloxane network on the surface of cerasomes.^43^ The drug release rate decreased as the number of triethoxysilyl headgroups in CFSLs increased.^32^ CFSLs **7** has one triethoxysilyl headgroup but three alkyl chains, which endows higher entrapment efficiency for hydrophobic drugs due to more compact bilayer structure.^43^ CFSLs **8** containing a disulfide bond provides glutathione‐responsiveness for controlled release.^44^ CFSLs **9** is a cationic amphiphilic CFSLs containing a quaternary ammonium group and was developed for gene transfection.^29^, ^45^ CFSLs **10** is a cholesteryl succinyl silane (CSS) which has selective antileukemia effects.^39^ CFSLs **11** bearing two triethoxysilyl headgroups and an integrated porphyrin was designed to afford more efficient PDT based on organized supramolecular assembly.^41^

The cerasome, first developed by Katagiri et al. in 1999,^31^ is now receiving substantial interests as a new type of therapeutic carrier in cancer therapy. Cerasome refers to an artificial bioinorganic hybrid liposome formed via in situ sol–gel processes by self‐assembly of cerasome‐forming silylated lipids (CFSLs) with a siloxane network that covalently coated on the bilayer membrane surface (Figure 1a). The cerasome is morphologically stable, infusible and can be encapsulated with hydrophilic, hydrophobic, and amphiphilic molecules for therapeutic delivery and biomedical imaging. Until now, various CFSLs have been synthesized (**Figure** **2**).^20^, ^32^, ^33^ A typical CFSL molecule consists of a trialkoxysilylated head and a hydrophobic dialkyl tail that are bridged through a head‐tail linkage (denoted as a “connector” in Figure 1a).^33^ The CFSL molecules can be simply converted to an amphiphilic structure by hydrolysis of the trialkoxysilylated head groups and formation of an artificial surface‐rigidified lipid bilayer in aqueous solution.

### 2.1 Preparation Approaches

Preparation methods for cerasomes are generically analogous to those for liposomes, and three common approaches are now used: 1) the ultrasonic dispersion method;^20^ 2) ethanol sol injection method; and 3) thin film hydration method. In the ultrasonic dispersion method,^20^ CFSLs and HCl were first mixed and agitated using a vortex mixer. The appearance of the dispersion turned into a milky white solution, which indicated the formation of multilamellar vesicles (MLVs). The MLV dispersion was then sonicated with a probe‐type sonicator in order to obtain cerasomes once the milky solution become slightly opalescent. It was noted that pH is an important factor for controlling hydrolysis rate and homogeneity in cerasome formation.^34^ Thus, a moderate acidic condition is preferred. Moreover, this method is usually applicable to water‐insoluble CFSLs, whereas the hydrolysis process is not needed if water‐soluble CFSLs were used.^20^ To overcome this limitation, Katigiri et al. improved the ultrasonic dispersion method by adding some modifications.^35^ In this procedure, an acidic ethanol solution of hydrolysed CFSLs was slowly injected into aqueous solution with a set pH followed by ultrasonication. In this case, cerasomes were formed via an independent self‐assembly process during the formation of siloxane network. In addition, a thin film hydration method in combination with sol–gel process and self‐assembly approach has also been reported.^36^ First, an acidic ethanol CFSL solution was added into chloroform, and the mixture was dried by a rotary evaporator to obtain a lipid thin film, followed by drying in a vacuum oven. The resulting dried product was hydrated with pure water or drug dissolved water via vortexing to form MLVs. The smaller cerasome size could be well controlled with further ultrasonication.

### 2.2 Morphological and Physiological Stability

The size of most cerasomes prepared from CFSLs was usually in the range of 20–200 nm. Owing to the presence of the shell cross‐linked network on the surface of cerasomes, superior biocompatibility, and physiological stability can be seen compared to silica nanoparticles and liposomes, respectively. Although pure cerasome‐incubated cells exhibited lower cell viability over that of conventional liposomes, hybrid cerasome doping by a certain percentage of natural lipids cannot only lessen cytotoxicity effects, but also have less influence on their morphological stability (Figure 1c). Moreover, the siloxane network coating on the cerasome surface allowed for a prolonged drug release profile in contrast to conventional liposomes.^37^ Cerasome drug release rates can be modulated by altering cerasome composition through incorporation of different dipalmitoylphosphatidylglycerol proportions.^36^ For example, an in vitro study demonstrated that insulin‐loaded cerasomes (using the dipalmitoylphosphatidylcholine (DPPC) as cosurfactants) exhibited several advantages as compared to injectable liposomal formulations, including more sustained insulin release, excellent long‐term storage stability, and lower drug leakage.^38^ These findings suggest that lipid organoalkoxysilanes have an important role such as providing structural building units to interplay the delicate balance between stability and drug release rate of the hybrid cerasomes.

Membrane stabilizers, typically cholesterol derivatives have also been engineered into cerasome building units to give enhanced stability and cancer therapy. For instance, Ma et al.^39^ synthesized a cholesteryl succinyl silane (CSS) (Figure 2, **NO. 10**) from a bioactive cholesteryl hemisuccinate (CS) precursor (an amphipathic lipid that inhibit C1498 and L1210 cancer cell growths),^40^ which underwent self‐assembly into cerasomes via the ethanol injection method, and doxorubicin (DOX), which loaded into hydrophobic cholesteryl succinyl bilayer membrane.^39^ The authors demonstrated that CSS cerasomes could selectively suppress leukemia cell proliferation without nonspecific cytotoxicity to normal blood cells. Moreover, DOX‐loaded CSS cerasomes further enhanced the therapeutic effect for treatment of leukemia.^39^ Photodynamic therapy (PDT) is a clinically approved, minimally invasive treatment for cancer. However, most photosensitizers suffer from poor chemical stability and photostability, and they tend to aggregate in aqueous solution, which decreases singlet oxygen quantum yield by exhausting energy through internal conversions. In this respect, Liang et al.^41^ developed a cerasomal photosensitizer prepared from self‐assembly of porphyrin‐conjugated organoalkoxysilylated lipids (PORSILs) bearing two triethoxysilyl groups (Figure 2, **NO. 11**). This hydrophobic double‐chain segment arrangement and siloxane network stabilization could effectively prevent photosensitizers from aggregation and prevent the undesirable loss of photosensitizers.^41^ The organized arrangement of porphyrins in cerasomal bilayer ensures efficient generation of singlet oxygen. They also demonstrated that the optical density bands of the porphyrin bilayer cerasomes (PBCs) can be readily adjusted by inserting metal ions into porphyrin moiety or by conjugating different photosensitizers.^41^ Furthermore, combination therapy could be achieved as PBCs are feasible for encapsulating therapeutic agents with high loading efficiency.

## 3 BIHBs

Bicelles are a fabulous category of lipid assemblies that have been extensively employed as membrane models for structural characterization and biophysicochemical study of membrane‐bound proteins.^46^ They consist of two types of phospholipids, which are long‐chain phospholipids that make up a planar region, and either detergent or short‐chain phospholipids that compose flanking rims. The bicelles' sizes (ranging from 15 to 100 nm diameter and ≈5 nm thickness) can be regulated by adjusting the lipid/detergent ratio, and the surface charge can be modulated by replacing neutral long‐chain lipids with phospholipids that have similar diacyl chain lengths but positively or negatively charged headgroups.^47^ It should be noted that bicelles are different from nanodiscs (NDs), which are engineered reconstituted high‐density lipoproteins mimics, NDs consist of discoidal phospholipid bilayers that are stabilized by a pair of membrane‐scaffolding proteins.^48^ Recently, bicelles have been increasingly explored as potential nanocarriers in several specific applications such as delivery of amphiphilic peptides, enhanced drug penetration for transdermal delivery, and large hydrophobic domains for delivery of water insoluble drugs (Figure 1d–g).

However, it is known that short‐chain lipids have distinct water solubility as compared to long‐chain phospholipids. Thus, decreased bicellar concentrations in samples may lower the stability of discoidal nanostructures as more short‐chain lipids are dissolved out from the bicelles.^49^ Besides, the morphological stability of bicelles can also be influenced by temperature,^50^ drug encapsulation and physiological milieu. Bioinorganic hybrid lipid‐based assemblies appear to provide a potential approach for improving conventional phospholipid bicelle stability. Yasuhara et al.^30^, ^51^ introduced BIHBs that consist of self‐assembling of long‐chain CFSLs (*N*,*N*‐dihexadecyl‐N^α^‐6‐[(3‐triethoxysilylpropyl)dimethylammonio]hexanoyl] alaninamide bromide) (Figure 2, **NO. 9**) and a short‐chain phospholipid, DHPC (1,2‐dihexanoyl‐sn‐glycero‐3‐phosphocholine). The stabilization procedure is approached via the sol–gel hydrolysis reaction, resulting in CFSL polycondensation. The authors elucidated that prepared BIHBs exhibit stable discoid morphology in aqueous solutions, even at temperatures beyond the phase transition (bicelle‐to‐vesicle transition) temperature of conventional phospholipid bicelles.^51^

## 4 Metallosomes

**Figure 3:**
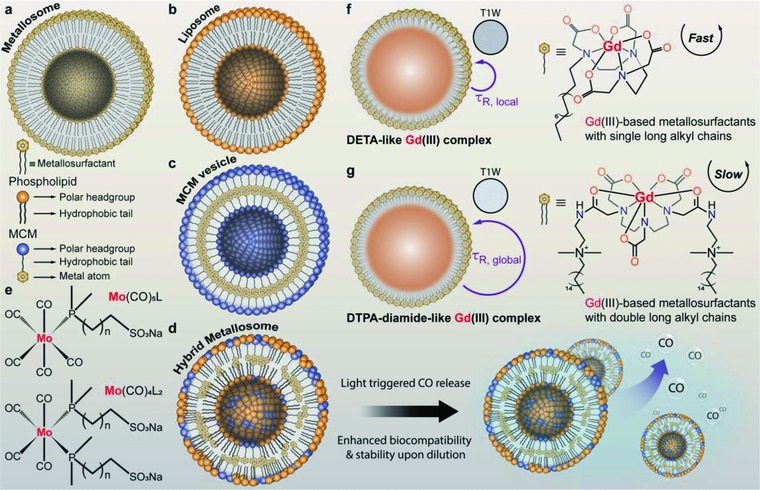
Metallsomes and their derivatives for therapeutics delivery and biomedical imaging. a) Comparative schematic illustration of the supramolecular structure of a typical metallosome, b) a pure liposome, c) a metallosome obtained with molybdenum carbonyl metallosurfactants (MCMs), and d) when they are mixed. e) The new type hybrid metallosome consists of phospholipids and MCMs, Mo(CO)_5_L or Mo(CO)_4_L_2_, which is stabilized inside the bilayer structure and releases the CO molecules upon the light stimulus (d).^54^, ^55^ f,g) Schematic depiction of Gd (III) complex formed with single or double long alkyl chains, and the kinematic characteristics of rotational motion among two types Gd (III)‐based metallosurfactants before and after incorporation into a polymer colloid.^56^, ^57^ Abbreviation: T1W: T1‐weighted contrast.

For development of more specific and individualized therapies for various diseases, nanotheranostics delivery systems have provided a transition platform by integrating different imaging strategies to improve the pharmacokinetics, potency, and safety profile of potential drugs.^52^ Lipid based self‐assembling amphiphiles are widely used in drug delivery systems and diagnostic imaging. However, simply mixing drugs and imaging agents into a nanosystem is not a rational strategy. In this scenario, one of the best ways to exploit these well‐organized and meaningful nanostructures for theranostics is to build supramolecular self‐assemblies consisting of lipid‐like functional metallosurfactants and phospholipids. These hybrid organometallic coordination‐assisted self‐assemblies are referred as metallosomes (**Figure** **3**a).

In general, double chain metallosurfactants are commonly used to prepare metallosomes. Alternatively, Garg et al.^53^ proposed a strategy to form manganese metallosomes (Mn‐somes) using a single‐chain cationic surfactant namely, cetyltrimethylammonium chloride, by controlling the stoichiometry of an embedded manganese (Mn) cationic ion. The obtained Mn‐somes displayed high encapsulation dye efficiency.^53^ This approach demonstrated the capability to modulate the structure of self‐assembled formulations based on cationic surfactants in addition to improve the stability.

## 5 BIHLAs for Controlled Release

Recently, increasing efforts have been directed at stimulus‐triggered drug delivery system based on cerasome innovations. The stimuli‐responsive systems hold switchable physicochemical properties when responding to specific stimuli including changes in the external physical environment, such as electromagnetic radiation, temperature, ultrasound, magnetic, and electric field, and/or internal biological cues that involve pH, redox, hypoxia, enzymes, nucleic acids, small biomolecules, and iron.^58^ Glutathione (GSH) is an important intracellular antioxidant that prevent cellular component damage caused by reactive oxygen species. It has been suggested that GSH tends to be elevated in breast, ovarian, head/neck, and/or lung cancers as compared to disease‐free tissues.^59^ In tumor cells, high GSH level can trigger disulfide bond (—S—S—) cleavage. In this regard, Zhou et al.^44^ developed a redox‐responsive cerasome that was composed of CFSLs bearing a cleavable —S—S— bond, and DOX, which was encapsulated as a model drug. In vitro results revealed that the presence of GSH in tumor cells triggered cerasome degradation via the —S—S— bond cleavage, followed by DOX release from cerasomes. Recently, a new approach has been investigated for in vivo treatment of cancer^60^ that uses high‐intensity‐focused‐ultrasound (HIFU), thermosensitive cerasomes (HTSCs) formed by CFSLs, and thermosensitive liposome forming lipids (such as 1,2‐dipalmitoyl‐sn‐glycero‐3‐phosphocholine‐/‐DPPC and 1,2‐distearoyl‐sn‐glycero‐3‐phosphoethanolamine‐*N*‐[methoxy(polyethylene glycol)‐2000]‐/‐DSPE‐PEG‐2000). The study showed that HTSCs exhibited desirable systemic circulation times with t_1⁄2_ > 8.50 h, which was much longer than non‐CFSL liposomes (≈1 h). Besides, HTSCs could selectively release drug molecules in the mildly hyperthermic tumor microenvironment (39–42 °C) due to the structural changes in temperature‐sensitive lipids.^61^ Furthermore, most drug payloads could rapidly liberate in 1 min upon HIFU sonication.^60^ Overall, the reported multiple stimuli‐responsive system provided precise and maximum therapeutic outcomes with minimal undesired side effects.

Aside from cerasomes, Lin et al.^62^ reported a pH sensitive BIHBs with high loading efficiency of hydrophobic drugs for in vitro and in vivo anticancer therapy. BIHBs were prepared from CFSLs and DHPC via the Bangham method in combination of sol–gel reaction and self‐assembly process.^62^ The resulting BIHBs exhibited good biocompatibility, long‐term stability in addition to superior cellular adhesion and internalization. Besides, BIHBs could not only be selectively taken up by tumor tissues, but also demonstrated increased drug release rates as pH values decreased in the tumor microenvironments.^62^ In another study, Lin et al.^23^ further optimized drug release rate by incorporation into BIHBs of DSPE‐PEG2000 phospholipids at certain molar percentages. As expected, the higher content of DSPE‐PEG2000 is correlated with drug release rate. Moreover, PEGylation of BIHBs resulted in enhanced biocompatibility, drug accumulation, cellular uptake, and improved antitumor efficacy.^23^ To achieve theranostics with BIHBs, Lin et al.^22^ coencapsulated DOX and indocyanine green (ICG) into the PEGylated BIHBs for synergetic chemo/photothermal therapy. NIR‐triggered drug release was observed due to the elevated drug release rate with increasing temperatures upon ICG‐induced photothermal effects.^22^

Recently, Barnadas‐Rodríguez and co‐workers developed new metallosurfactants with alternative designs.^54^, ^55^, ^63^ Usually, the metal atom form the polar headgroup of the metallosurfactants (Figure 3a), while, in this design, metallic fragments are embedded in the hydrophobic end of the surfactants (Figure 3c). A CO‐delivery system based on supramolecular assembling of molybdenum (Mo) carbonyl metallosurfactants was developed.^55^ These compounds demonstrated slow CO‐release, which could be potentially useful in therapeutic applications. However, instability maybe observed when these suspensions are diluted, which is limited for in vivo applications.^54^ To this end, they incorporated bilayer‐forming metallosurfactants into the phospholipid bilayer in order to obtain stable hybrid metallosomes upon dilution (Figure 3d). Notably, the new nanosystems also demonstrated a dramatic cytotoxicity mitigation as compare to free metallosurfactants.^54^ Due to these improvements, this novel strategy could be adapted to prepare other low toxicity, metal‐based therapeutic metallosomes.

## 6 BIHLAs for Targeting Delivery

Mitochondrial malfunction is centrally involved in various chronic diseases including Alzheimer's disease, Parkinson's disease (PD), obesity, diabetes, and cancer.^64^ Wang et al.^65^ described a 3‐aminopropyl triethoxysilane (APS) modified‐(5‐carboxypentyl) triphenylphosphoniumbromide (TPP) decorated cerasome for mitochondrial‐targeted DOX delivery. A TPP targeting moiety was covalently grafted onto the surface of cerasomes through the hydrolytic Si‐OH condensation between the CFSL head and APS–TPP. While the drug release rate was modulated by altering the lipid composition, an in vitro study confirmed the TPP mitochondria targeting efficacy of TPP–CER–DOX based on quantitative analysis of the mitochondrial transmembrane potential reduction.^65^ Over the past decade, monoclonal antibodies targeting specific cell–surface antigens that were overexpressed in different tumor types have been substantially exploited for targeting cancer therapy.^66^ Leung et al. developed anti‐epidermal growth factor receptors monoclonal antibodies (anti‐EGFR mAbs) functionalized with liposomal hybrid cerasomes, which target EGFR on cell surfaces.^67^ In this study, the cerasomes formulation consisted of CFSLs, cholesterol, *N*‐(7‐nitrobenz‐2‐oxa‐1,3‐diazol‐4‐yl)‐1,2‐dihexadecanoyl‐sn‐glycero‐3‐phosphoethanolamide (NBD‐DPPE), and DSPE‐PEG (2000) Maleimide (MAL‐DSPE‐PEG) at a 14.2:9.4:0.2:1 molar ratio.^67^ Anti‐EGFR mAbs were conjugated to MAL‐DSPE‐PEG via maleimide‐thiol coupling chemistry, resulting in immunocerasomes for targeting A431 cell treatment.^67^ An in vitro study found that immunocerasomes exhibited similar anti‐proliferative effects compared to free anti‐EGFR mAbs. In addition, the presence of serum in a cell culture medium greatly promoted the endocytosis of immunocerasomes but inhibited cellular uptake of cerasomes by A431 cells,^67^ suggesting high‐affinity immunocerasome targeting effects in cells overexpressing EGFR. In another example, cryptotanshinone‐loaded cerasomes (CTS‐CS) capable of treating acne were reported by Zuo et al.^68^ In this study, CTS was used as a model drug for CTS‐CS fabrication via the ethanol injection method. In vitro and in vivo investigations have revealed that the CTS‐CS gel exhibited rapid drug permeation rates and significant dermal accumulation, which may be useful for topical acne therapy.^68^ In addition, compared with ordinary formulations, CTS‐CS showed improved bioavailability but minimum transdermal penetration into plasma, which lowered the undesirable effects.^68^ These results indicate that cerasomes could be used as an effective transdermal‐delivery system for topical treatment of skin diseases.

## 7 BIHLAs for Imaging Contrast and Theranostics

Liposomal hybrid cerasomes have been demonstrated as a potential theranostic system by encapsulating inorganic contrast agents such as superparamagnetic iron oxide (SPIO) nanoparticles and chemotherapeutics^69^, ^70^ or by dissolving hydrophobic chemotherapeutics in the lipid membrane.^71^ Ma et al.^70^ reported theranostic gold nanoshell‐coated CSS nanomicelles (CDF‐Au‐shell nanomicelles), loaded with DOX and SPIO nanoparticles for magnetic resonance imaging (MRI)‐guided photothermal ablation combined with magnetic‐targeted and light‐triggered drug release for cancer therapy. CDF‐Au‐shell nanomicelles were prepared via a multistep procedure, in which DOX and SPIO nanoparticles were first loaded into self‐assembled CSSs and then were self‐rigidified to form CDF nanomicelles via in situ sol–gel processes. Negatively charged gold seeds were then deposited onto the surface of CDF nanomicelles, and an outer‐layered gold nanoshell was formed via nuclear growth.^70^ In another study, Jing et al. described theranostic ICG‐loaded CSS cerasomes by introducing DSPE‐PEG2000‐DOTA with a chelating radioisotope of ^177^Lu.^72^ The in vivo NIR fluorescence and SPECT/CT imaging guided biodistribution of ICG@DPDCs‐^177^Lu demonstrated selective accumulation at the tumor site after intravenous administration,^72^ resulting in maximal photothermal effect at an ideal time during the therapeutic window due to the real‐time multi‐image monitoring.^72^ Remarkably, physiologically stable cerasomes protected bioactive payloads from degradation, aggregation, and rapid elimination from the body, which provided an excellent platform for theranostic application. A recent cerasome application has been utilized for gene delivery. Li et al.^73^ reported PEGylated cationic cerasomes (PCCs) consisting of CFSLs, a PEGylated lipid, and a cationic lipid with a hydroxyl group. Cy5‐siRNA was loaded onto the PCC surface, and an in vitro study showed that PCCs could enhance siRNA delivery, and the presence of hydroxyl groups on the PCC surface could promote siRNA escape from the lysosome.^73^ An in vivo study found that PCCs could effectively deliver siRNAs to liver and suppress the target gene. Cerasomes are also being engineered for ultrasound contrast imaging. Zhang et al.^74^ described a hard‐template approach for perfluoropropane‐loaded cerasomal microbubble (PLCM) formation, which combined structure‐based features of both lipid and silica microbubbles. In this report, the PLCMs were prepared from CFSL deposition onto functionalized CaCO_3_ microspheres followed by template core removal and mild infusion of perfluoropropane.^74^ The resulting PLCMs demonstrated high colloidal stability, tunable and uniform size distribution, low interfacial tension, and good biocompatibility, which resulted in excellent echoing characteristics and prolonged ultrasound contrast imaging as compared with commercially used SonoVue. Most recently, Du et al.^75^ described an MRI/NIRF imaging‐assisted in vivo evaluation of EGFR‐targeted liposomal nanohybrid cerasomes in colorectal cancer treatment that was based on PD‐L1 immunotherapy in combination with chemotherapy. The cerasomes were prepared by self‐assembly of CFSLs and DSPE‐conjugated‐ IRDye800CW, Gd‐DOTA, and PD‐L1 antibodies.^75^ Paclitaxel molecules were loaded into the bilayer hydrophobic domain of cerasomes. Dual‐mode imaging enabled noninvasive monitoring of cerasome' in vivo distribution and found preferential cerasome accumulation at tumor sites.^75^ Significantly, the cerasomes also played an adjuvant role in enhancing paclitaxel efficacy. The authors provided a promising theranostic nanoplatform based on cerasomes that targeted immune checkpoints^75^ and offered potential opportunities to optimize therapeutic effects for clinical translation.

Metallosomes that integrate with functional metal ions can be specifically designed to boost theranostic effects, for example, enhancement of MRI contrast using stable chelates of Gd (III) can be demonstrated. It is known that self‐assembled micelles or vesicles of amphiphilic Gd (III) complexes can increase their relaxivity by hindering molecular in water.^76^ However, self‐assembling systems are colloidally unstable for dilution under physiological conditions. To address this issue, Gong et al.^57^ reported high relaxivity metallo‐colloids (up to 240% enhancement in relaxivity as compare to other small molecular counterparts) using Gd (III)‐based lipid‐like metallosurfactants (Figure 3f), which was approached through miniemulsion of mixed cetyl alcohol, styrene, divinylbenzene, metallosurfactants, and cosurfactants, followed by polymerization process with addition of oil‐soluble initiator azobisisobutyronitrile (AIBN). The obtained Gd (III)‐based colloids are morphologically stable to dilution and showed dramatic improvements in relaxivity.^57^ Furthermore, such techniques could also be applied for development of multimodal imaging agents for future in vivo applications. Although enhanced *T*_1_‐weighted contrast and morphological stability have been achieved, these metallosurfactants have poor dispersibility, and cosurfactants are required to promote the formation of miniemulsions.^56^ Moreover, one of the coordination sites of the polychelating ligands in these metallosurfactants was occupied by the long alkyl chain, resulting in limited dynamic stability of the Gd (III) complexes.^77^ By taking this into account, Chen et al. developed a DTPA‐diamide‐like Gd (III) complex functionalized with double quaternary‐ammonium‐containing long alkyl chains to address both the dispersibility and dynamic stability issues (Figure 3g),^56^ which permitted self‐assembly into micelles and finally formation of a polymeric nanocolloids via miniemulsion polymerization. The resulting Gd (III)‐containing nanocolloids showed effective tumbling restrictions in Gd (III) complexes, leading to improved relaxivity.^56^

A recent advance in metallosome‐based theranostics has been reported by Moghaddam et al.^78^ A theranostic organometallic nanoassembly consisted of Gd/Eu‐DTPA amphiphiles and α‐Flag antibody‐conjugated EDTA‐amphiphiles were prepared to achieve active targeted *T*_1_‐weighted MR images and Gd‐neutron capture therapy. The authors found that amphiphilic molecular structure and modulation of Gd content could distinctly control the size and structure of nanoassemblies, ranging from micelles to liposomes, hexosomes, cubosomes, and multilayered nanospheres.^78^ These nanoassemblies showed increased relaxivity and low cytotoxicity in addition to versatility in loading various radioisotopic and luminescent metal ions. Later, a comparable study was described by Gupta et al.^79^ In this study, Gd (III) chelated‐DTPA‐monophytanyl (Gd‐DTPA‐MP) amphiphiles and phytantriol (PT) self‐assembly could lead to the formation of cubosomal and liposomal nanostructures by varying the Gd‐DTPA‐MP/PT molar ratio. Both nanostructures exhibited enhanced relaxivities compared to pure Gd‐DTPA‐MP dispersion,^79^ which might imply potential theranostic in vivo applications.

## 8 Perspectives for Clinical Translation

Despite the potential advantages of using BIHLAs, some on‐going challenges need to be acknowledged and resolved. With regard to cerasomes and bicelles, the acquired high mechanical stability could be a two‐edged sword for drug delivery. Although the drug release rate can be regulated by adjusting the phospholipid/CFSLs ratio, the ideal in vivo drug release profile may be difficult to achieve due to the interplay between stability control and desirable release rate. In addition, reproducibility of maintaining specific drug release rates could vary from bench to bench and time to time. Stimuli‐responsive strategies need to be involved for more effective control of drug release. In addition, the relatively broad size distribution of cerasomes and bicelles is an important issue that can largely affect their pharmacokinetics and biodistribution. Extrusion processes or removable hard‐template approaches may be used to form homogeneous size distribution for in vivo applications. Further investigations for clinical translation should focus on the evaluation of in vitro and in vivo metabolic pathways of CFSL‐based assemblies.

Metallosome application in drug delivery has been less explored, and most studies on metallosomes at present focus on in vitro proof‐of‐concept. Systematic metallosome safety studies in vivo are a major challenge for practical applications. For example, over time there have been increasing evidence suggesting that frequent exposure to Gd (III)‐based contrast agents is associated with varying risk of developing nephrogenic systemic fibrosis and mainly affects patients with kidney diseases.^80^ However, the development of lanthanide coordination chemistry leads to the improved image quality and greater dose efficiency, which in turn reduces the patients exposure to lanthanides. Regardless of potential toxicity, the concurrent diagnostic and therapeutic approaches can effectively accelerate in vivo and prognostic evaluations during preclinical development, which will ultimately be beneficial to human patients by providing accurate predictions of therapeutic outcomes.

## Conflict of Interest

The authors declare no conflict of interest.
